# A De Novo sSMC (22) Characterized by High-Resolution Chromosome Microarray Analysis in a Chinese Boy with Cat-Eye Syndrome

**DOI:** 10.1155/2021/8824184

**Published:** 2021-02-27

**Authors:** Jinjie Li, Yue Zhang, Yanjun Diao, Rui Li, Liqing Jiang, Lei Zhou, Jiayun Liu, Weixun Duan, Liu Yang

**Affiliations:** ^1^Department of Laboratory Medicine, Xijing Hospital, Air Force Military Medical University, Xi' an 710032, China; ^2^Department of Cardiovascular Surgery, Xijing Hospital, Air Force Military Medical University, Xi'an 710032, China

## Abstract

We report a 15-year-old boy with cat-eye syndrome (CES) without short stature or intellectual disorder. The boy was confirmed by cytogenetic and high-resolution chromosome microarray analysis (CMA). The G-banding karyotype confirmed the de novo of the patient. Also, the CMA result showed 1.76 Mb tetrasomy of proximal 22Q11.1 ⟶ 22Q11.21 consistent with CES {arr22q11.1q11.21 (16,888,899–18,644,241) X4}, a typical small type I CES chromosome. The patient has many of the basic characteristics of CES; however, he is taller than his peers instead of shorter. It is rarely reported in the past since short stature is a common feature of this syndrome. Furthermore, the boy has no intellectual disorder and attends a normal school since he was six-year-old. What bothered him most were recurrent respiratory infections, retromicrognathia, and heart defects.

## 1. Introduction

Cat-eye syndrome (OMIM 115470) is a rare chromosomal syndrome associated with a small supernumerary marker chromosome (sSMC), which is derived from duplicated regions of 22pter-22q11.2. The estimated prevalence of CES is 1 : 50,000–1:150,000 [1]. The syndrome was named “cat eye” due to the typical ophthalmological finding of vertical colobomas in few patients. The most common symptoms are preauricular tags and pits, anal atresia, and coloboma of the iris [2, 3]. However, clinical features reveal an extreme phenotypic variability, including variable congenital heart defects and kidney and skeletal abnormalities, which may or may not be associated with variable mental deficiency [2, 3].

There are about 100 patients reported till now with CES around the world to our knowledge. Here, we report a fifteen-year-old boy with clinical features of CES, confirmed by cytogenetic and high-resolution chromosome microarray analysis (CMA) indicating the breakpoints of the duplication as well as the gene content.

## 2. Case Presentation

**Figure 1:**
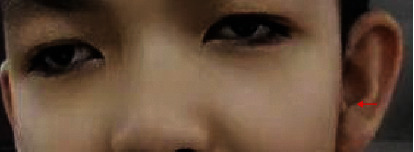
Eyes features of the patient at the age of 15. Note the downslanting palpebral fissures and strabismus.

When the boy came to our hospital for a bradycardia, he was 15-year-old. He was the first child of nonconsanguineous and healthy parents. Family history was unremarkable. However, the patient's mother worked at a gas station before the pregnancy, and she moved into a newly renovated house around the pregnancy. Besides, she had been knocked down by a child during the first trimester and treated with utrogestan. The proband was born at 36 weeks gestation with a birth weight of 3400 g. At birth, he was diagnosed with atrial septal and ventricular septal defect resolved by cardiac surgery after one month. He also accepted surgical intervention for subdural effusion and later for inguinal hernia and congenital coloboma of choroid of his right eye. His craniofacial features included long face, broad forehead, downslanting palpebral fissures (Figure 1), strabismus, and bilateral preauricular pits. No hearing loss was noticed. He sometimes ate with eye tears when he swallowed too fast because of the retromicrognathia. Musculoskeletal abnormalities were the following: kyphoscoliosis, torticollis, longer fingers and toes, rich palm drawing, and pes planus. Especially, he is taller than his peers instead of shorter, his height was 180 cm, and weight was about 45 kg at the age of 15. And he had no obvious mental disability either. However, he probably had an attention deficit disorder according to his mother's dictation without doing a neuropsychiatric examination. From the neonatal period to the present age, the main health problems of the propositus were recurrent respiratory infections.

## 3. Methods

### 3.1. Cytogenetic Analyses

The cytogenetic analyses of the patient and his parents with cleft lip and palate were made on G-banded chromosomes obtained from 72-hour lymphocyte cultures.

### 3.2. High-Resolution Chromosome Microarray Analysis

To ascertain the chromosomal origin and segmental composition of the sSMC, a high-resolution genomic scan using Affymetrix CytoScan 750K affay was performed in the patient. Analysis of the arrays was performed using Genotyping Console v4.0 and ChAS v1.2 software.

All samples were taken after an informed consent had been signed.

## 4. Results

### 4.1. Cytogenetic Analyses

**Figure 2:**
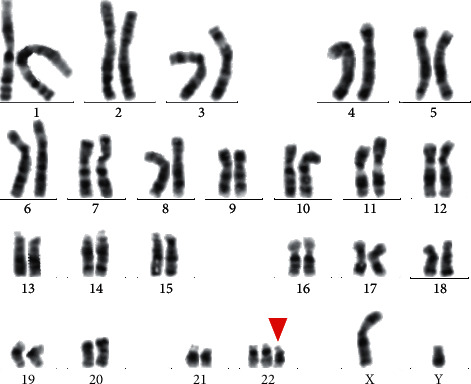
G-banding metaphase and karyotype of patient.

The patient's G-banded karyotype showed a male karyotype 47, XY, +mar. ish idic (22) (q11.2) (20) (Figure 2). However, his father and mother showed a normal male karyotype and a normal female karyotype, respectively (Figure 2S), thus confirming the de novo appearance of the marker chromosome in the proband.

### 4.2. High-Resolution Chromosome Microarray Analysis

**Figure 3:**
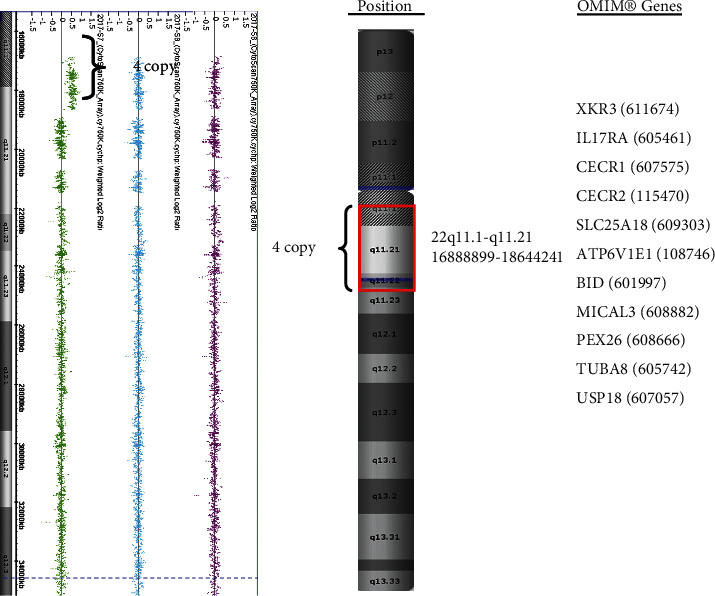
Results of high-resolution chromosome microarray analysis.

The result showed 1.76 Mb tetrasomy of proximal 22Q11.1⟶22Q11.21 consistent with cat eye syndrome {arr22q11.1q11.21 (16,888, 899–18,644, 241) X 4} (Figure 3). There were 4 copies of 12 genes, including *XKR3*, *IL17RA*, *CECR1*, *CECR2*, *SLC25A18*, *ATP6V1E1*, *BID*, *MICAL3*, *PEX26*, *TUBA8*, and *USP18* (Table 1). The four-copy gains of this region are typically associated with a supernumerary bisatellited marker. However, the present approach was not sufficient to define the parental chromosomal origin of the sSMC.

## 5. Discussion

Cat-eye syndrome is a rare malformation syndrome which was first reported in 1965 by Schachenmann [5]. The ratio of males to females was nearly 1 : 3 according to the reported cases. We present a 15-year-old boy with many of the basic characteristics of CES as preauricular pits and tags, inferior coloboma of the iris/choroid and retia, retromicrognathia, and downslanting palpebral fissures, but lacking anal atresia or mental retardation [6–9]. In particular, he suffered from congenital heart disease and came to the hospital for a bradycardia.

The patient was referred for genetic evaluation due to the multiple manifestations. Firstly, a routine G-banding karyotype analysis was performed for the proband and his parents. Karyotype analysis of the patient revealed a bisatellited mosaic SMC (Figure 2), and the parents showed normal karyotype in both, thus confirming the de novo of the proband. Then, a chromosome microarray analysis (CMA) was done using Affymetrix CytoScan 750K affay for the boy. The result showed 1.76 Mb tetrasomy of proximal 22Q11.1⟶22Q11.21. The four-copy gains of this region contain 11 genes (Table 1 and Figure 3). Most of them are dosage sensitive, so their function could be affected by extra copies, especially *CECR2*, the CES-related gene. Comparing to conventional cytogenetic analysis methods, the whole genome microarray scanning technique is of high resolution, high throughput, and high accuracy, which facilitates accurately screening out pathogenic copy number variations and genes and investigating karyotype-phenotype correlation. So the whole genome microarray scanning technique can serve as a useful complement for G-banding to be used in the clinical cytogenetic diagnosis [10].

According to McTaggart et al.'s classification, CES chromosomes fall into two types based on the location of the breakpoints required to generate them. The small type I CES is symmetrical with both breakpoints located with the proximal interval, and the larger type II is either asymmetrical, with one breakpoint located in each of the two intervals, or symmetrical with both breakpoints located in the distal interval [11, 12]. Our proband has a de novo SMC 22q11.1-q22.2 duplication of 1.76 Mb, containing two copies of the CECR rendering the patient tetrasomic for this region. The breakpoints of the duplication, characterized by CMA, begin at the repeated sequences within the chromosome 22 centromere (22q11.1) to the low copy repeat block A (LCR22-2:16,888,899–18,644,241, hg19) (22q11.21), indicating this to be a small type I CES chromosome [13–15].

CES syndrome shows phenotypic variability, and correlation of clinical severity has neither been found with the size of the marker chromosome nor with the extent of mosaicism in mosaic CES. Our patient manifested two of the three typical characteristics : an iris coloboma and ear anomalies. He does not suffer the anal malformation. Only 41.6% of the reported more than 100 CES patients presented with all the three main characteristics features. The boy in this paper now lives almost a normal life, and he is good at playing drums actually. However, he should pay attention to the recurrent respiratory infections, retromicrognathia, and heart defects, especially the retromicrognathia, which affects his dysphagia.

## 6. Conclusion

Here, we reported a CES case who had a de novo SMC 22q11.1-q22.2 duplication of 1.76 Mb by the whole genome microarray scanning technique. The differences from other cases were that the patient had no obvious mental disability, and he was taller than his peers instead of shorter. It has enriched the phenotypes of the reported cases.

## Figures and Tables

**Table 1:** Known protein coding genes and their function in the 22q11.1q11.21 (16,888,899–18,644,241), 1.76 Mb duplicated CES region [4].

Gene	Gene location^a^	Name	%HI^b^	Function
*ATP6V1E1*	18,074,903–18,111,588	Lysosomal ATPase, H1 transporting, 31 kDa, V1 subunit E1	48.8	V-ATPase responsible for acidifying a variety of intracellular compartments
*BID*	18,216,906–18,256,808	BH3 interacting domain death agonist	89.1	Member of the BCL-2 family of cell death regulators
*CECR1*	17,660,192–17,690,779	CES chromosome region, candidate 1	36.7	Regulates the concentration of extracellular adenosine
*CECR2*	17,956,630–18,033,845	CES chromosome region, candidate 2	52.7	Putative transcriptional coactivator
*IL17RA*	17,565,849–17,596,584	Interleukin 17 receptor A	37.5	Proinflammatory cytokine
*MICAL3*	18,270,416–18,507,325	Microtubule-associated monooxygenase calponin and LIM domain	76.3	Cytoplasmic semaphoring/plexin signaling molecule; expressed in motor neurons
*PEX26*	18,560,686–18,573,797	Peroxisome biogenesis factor 26	98	Required for protein import into peroxisomes
*SLC25A18*	18,043,183–18,073,647	Solute carrier family 25 (mitochondrial carrier)	80.9	Transport of glutamate across the inner mitochondrial membrane
*XKR3*	17,264,306–17,302,584	XK, Kell blood group complex subunit-related family	ND	Component of the XK/Kell blood group system
*USP18*	18,632,758–18,660,162	Ubiquitin specific peptidase 18	ND	Member of the de-ubiquitinating protease family of enzymes
*TUBA8*	18,593,453–18,614,498	Alpha tubulin isoform 8	ND	Associated with polymicrogyria and optic nerve hypoplasia

^a^University of California Santa Cruz (UCSC) database (http://genome.ucsc.edu/), UCSC Genome Browser, human genome 19/build 37. ^b^Haploinsufficiency Score (HI index): high ranks (e.g., 0–10%) indicate a gene is more likely to exhibit haploinsufficiency and low ranks (e.g., 90–100%) indicate a gene is more likely to NOT exhibit haploinsufficiency (Huang et al., 2010). CES, cat-eye syndrome; HI, haploinsufficiency score; ND, not determined.

## Supplementary Materials

Supplementary Materials The Supplementary Materials contain the G-banding metaphase and karyotype of patient's parents.
