# Supplementary figures and images for: A De Novo sSMC (22) Characterized by High-Resolution Chromosome Microarray Analysis in a Chinese Boy with Cat-Eye Syndrome
Source: Case Rep Genet. 2021 Feb 27;2021:8824184. doi: 10.1155/2021/8824184 (PMC7937470; doi:10.1155/2021/8824184)

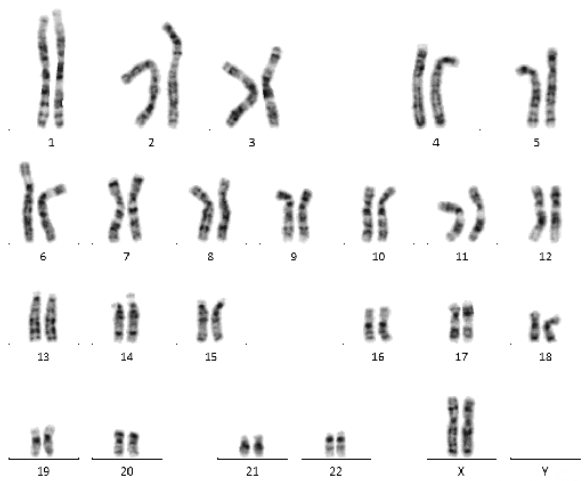


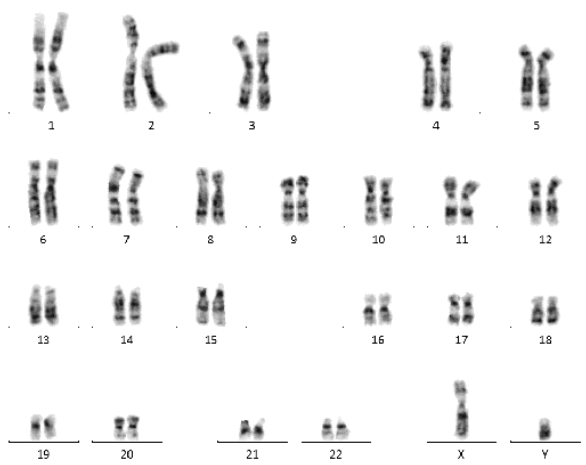


Figure 2S. G-banding metaphase and karyotype of patient’s parents

Supplement: Supplementary Materials — The Supplementary Materials contain the G-banding metaphase and karyotype of patient's parents. [file 8824184.f1.docx]
